# Anthocyanins from *Aristotelia chilensis* Prevent Olanzapine-Induced Hepatic-Lipid Accumulation but Not Insulin Resistance in Skeletal Muscle Cells

**DOI:** 10.3390/molecules26206149

**Published:** 2021-10-12

**Authors:** Andrea del Campo, Catalina Salamanca, Angelo Fajardo, Francisco Díaz-Castro, Catalina Bustos, Camila Calfío, Rodrigo Troncoso, Edgar R. Pastene-Navarrete, Claudio Acuna-Castillo, Luis A. Milla, Carlos A. Villarroel, Francisco A. Cubillos, Mario Aranda, Leonel E. Rojo

**Affiliations:** 1 Departamento de Biología, Facultad de Química y Biología, Universidad de Santiago de Chile, Santiago 9170002, Chile; catalina.salamanca@usach.cl (C.S.); angelo.fajardo@usach.cl (A.F.); catalina.bustos@usach.cl (C.B.); camila.calfio@usach.cl (C.C.); claudio.acuna@usach.cl (C.A.-C.); 2 Laboratorio de Fisiología y Bioenergética Celular, Escuela de Química y Farmacia, Facultad de Química y de Farmacia, Pontificia Universidad Católica de Chile, Santiago 7820436, Chile; 3 Laboratorio de Investigación en Nutrición y Actividad Física, Instituto de Nutrición y Tecnología de los Alimentos (INTA), Universidad de Chile, Santiago 7830490, Chile; fdiaz@inta.uchile.cl (F.D.-C.); rtroncoso@inta.uchile.cl (R.T.); 4 Advanced Center for Chronic Diseases (ACCDiS), Universidad de Chile, Santiago 8380492, Chile; 5 Laboratorio de Síntesis y Biotransformación de Productos Naturales, Departamento de Ciencias Básicas, Facultad de Ciencias, Universidad del Bío-Bío, Chillán 4081112, Chile; epastene@ubiobio.cl; 6 Escuela de Medicina, Universidad de Santiago de Chile, CIBAP, Obispo Umaña 050, Santiago 9170201, Chile; luis.milla@usach.cl; 7 ANID-Programa Iniciativa Científica Milenio-Instituto Milenio de Biología Integrativa (iBio), General del Canto 50, Providencia, Santiago 7500565, Chile; carlos.villarroel.f@usach.cl (C.A.V.); francisco.cubillos.r@usach.cl (F.A.C.); 8 Laboratorio Interacciones Insecto-Planta, Instituto de Ciencias Biológicas, Universidad de Talca, Talca 3460000, Chile; 9 Departamento de Biología, Facultad de Química y Biología, Universidad de Santiago de Chile, Santiago 9170002, Chile; 10 Laboratorio de Investigación en Fármacos y Alimentos, Departamento de Farmacia, Facultad de Química y de Farmacia, Pontificia Universidad Católica de Chile, Santiago 7820436, Chile; mario.aranda@uc.cl

**Keywords:** anthocyanins, insulin resistance, lipid accumulation, akt phosphorylation, mitochondrial oxygen consumption

## Abstract

Type 2 diabetes and obesity are major problems worldwide and dietary polyphenols have shown efficacy to ameliorate signs of these diseases. Anthocyanins from berries display potent antioxidants and protect against weight gain and insulin resistance in different models of diet-induced metabolic syndrome. Olanzapine is known to induce an accelerated form of metabolic syndrome. Due to the aforementioned, we evaluated whether delphinidin-3,5-*O*-diglucoside (DG) and delphinidin-3-*O*-sambubioside-5-*O*-glucoside (DS), two potent antidiabetic anthocyanins isolated from *Aristotelia chilensis* fruit, could prevent olanzapine-induced steatosis and insulin resistance in liver and skeletal muscle cells, respectively. HepG2 liver cells and L6 skeletal muscle cells were co-incubated with DG 50 μg/mL or DS 50 μg/mL plus olanzapine 50 μg/mL. Lipid accumulation was determined in HepG2 cells while the expression of p-Akt as a key regulator of the insulin-activated signaling pathways, mitochondrial function, and glucose uptake was assessed in L6 cells. DS and DG prevented olanzapine-induced lipid accumulation in liver cells. However, insulin signaling impairment induced by olanzapine in L6 cells was not rescued by DS and DG. Thus, anthocyanins modulate lipid metabolism, which is a relevant factor in hepatic tissue, but do not significantly influence skeletal muscle, where a potent antioxidant effect of olanzapine was found.

## 1. Introduction

A particularly severe form of type 2 diabetes (T2D) is induced by second-generation antipsychotics (SGAs) [1,2,3], such as olanzapine (OLZ). This has become a matter of public health interest [4] since it turns into full-blown diabetes within 3–5 months after initiation of pharmacotherapy [2,3], and billions of people are exposed to these substances worldwide. The main cardiometabolic side effects of SGAs are insulin resistance, type 2 diabetes, and obesity, for which efficacious prophylactic interventions are not yet available [2,3]. The mechanisms underlying this accelerated form of diabetes are still a matter of debate. However, several hypotheses have been proposed, namely (i) changes in markers of adipocytes’ differentiation [5], (ii) induction of lipid biosynthesis in liver and adipose tissue [6,7], (iii) overexpression of SREBP (protein binding to the sterol regulatory element) in adipocytes and hepatocytes [8,9,10], and (iv) hyperphagia induced by alterations in signals that participate in appetite regulation [11]. Given the clinical evidence on the prevalence of metabolic side effects of SGA, several pharmacological and non-pharmacological interventions have been studied to prevent this form of metabolic syndrome, but none of these interventions have shown efficacy [12].

Anthocyanins are one of the major groups of natural pigments and have been extensively studied in the context of food technology applications and human nutrition [13,14,15,16]. Numerous basic and clinical studies have demonstrated that anthocyanins, and other polyphenols from various botanical sources, are efficacious in ameliorating diabetes, obesity, and metabolic syndrome [17,18]. For example, Anderson et al. demonstrated that a cinnamon extract could lower fasting glucose, LDL, and HDL plasma levels [6]. An improvement in insulin resistance was also achieved after consuming anthocyanins-rich cranberries and pomegranate juices [5,19]. Recent evidence suggests that polyphenols from the anthocyanin family have anti-inflammatory, antidiabetic, antiobesity, and cardioprotective effects [7,8,9,17]. For example, anthocyanins from black soybeans increased insulin sensitivity by activating AMPK in skeletal muscle and liver in a type 2 diabetic mouse model [10]. AMPK regulates glucose and lipids metabolism in the liver and is inhibited by pharmacological therapy with OLZ, which is thought to contribute to SGAs-induced hepato-steatosis [11]. In this same study, the authors concluded that an anthocyanins-rich formula improves hyperglycemia and insulin resistance in obese mice fed with a high-fat diet by modulating glucose metabolism in the liver and skeletal muscle [10]. Resveratrol and green tea have shown efficacy in decreasing weight gain induced by OLZ in rodents [20]. This evidence is important since OLZ-induced diabetes is a severe, life-threatening condition with very limited prophylactic interventions. We have previously reported that delphinidin-3-sambubioside-5-glucoside (DS) displays insulin-like effects in skeletal muscle and liver and ameliorates insulin resistance in high-fat diet-induced diabetes [18]. Another study confirmed these findings, showing that an extract rich in delphinidins reduced postprandial blood glucose increase in individuals with impaired glucose regulation [21]. In addition, a clinical study demonstrated that anthocyanins-rich extract from *Aristotelia chilensis* (Molina) Stuntz (Elaeocarpaceae) decreased postprandial plasma levels of glucose and insulin in patients with impaired glucose tolerance, suggesting its potential use in pre-diabetic patients [22]. Maqui anthocyanins also improve oxidative status in [23] in healthy adults, overweight adults, and adult smokers. Antidiabetic anthocyanins from Maqui fruit are mainly glycosylated forms of delphinidin [18]. Notably, DS from Maqui has been reported as an insulin-like molecule in liver and muscle cells [18,24]. Thus, the published literature shows strong evidence supporting the pharmacological effects of anthocyanins from *A. chilensis* fruit against diabetes and insulin resistance [12,18,25]. It has already been proven that polyphenols from various sources can positively impact diabetes, obesity, and metabolic syndrome. We aimed to evaluate whether two anthocyanins with antidiabetic properties isolated from Maqui [18] can prevent OLZ-induced steatosis in HepG2 liver cells and insulin resistance in L6 skeletal muscle cells.

## 2. Results

### 2.1. Anthocyanins Extraction and Characterization

**Figure 1:**
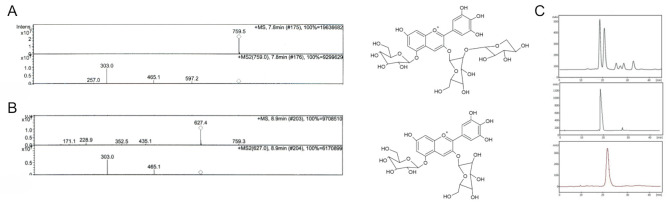
Maqui anthocyanins DS and DG characterization by mass spectroscopy. (**A**) MS spectrum of delphinidin 3-*O*-sambubioside-5-*O*-glucoside isolated by CPC: *m*/*z* 759.5 MS-MS *m*/*z* 597.2, 465.1, 303. (**B**) MS-MS spectrum of delphinidin 3,5-*O*-diglucoside isolated by CPC: *m*/*z* 627: MS-MS *m*/*z* 465, 303. (**C**) LC/UV chromatogram crude extract (upper panel), DG (middle panel), DS (lower panel) at λ 520 nm.

Fresh Maqui fruit (500 g) was used for the extraction of DS and DG. Amberlite XAD-7 clean-up of crude extract was necessary to remove sugars, proteins, and other organic non-phenolic compounds. An aliquot (500 mg) of purified crude extract of *A. chilensis* fruits was subjected to centrifugal partition chromatography. As described in Section 4.2, this CPC procedure enabled the one-step purification of 58 mg of DS and 27 mg of DG with purities of 97.6 and 98.8%, respectively. Purity and identity were verified by MS/MS analyses, as shown in Figure 1. DS showed a molecular ion at *m*/*z* 759.5 with MS/MS fragments at *m*/*z* 597.2 and 465.1, which correspond to the loss of hexose moiety (162 u) and hexose and pentose (162 + 132 u), respectively. Additionally, the delphinidin aglycon signal was also observed at *m*/*z* 303.0. DG showed a molecular ion at *m*/*z* 627.4 with MS/MS fragments at *m*/*z* 465.1 and 303.0 corresponding to one hexose lost (162 u) and the delphinidin aglycon. These data are concordant with the fragmentation patterns of DG and DS reported previously [18]. 

### 2.2. Metabolic Effects of Olanzapine in Liver Cells

**Figure 2:**
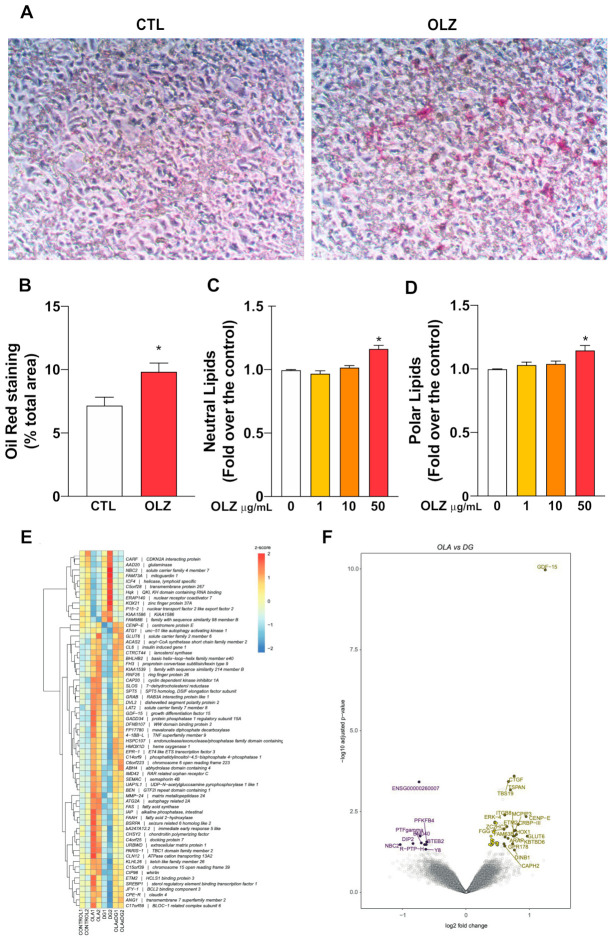
Effect of olanzapine on HepG2 cells’ lipid content. (**A**) Representative images of control and OLZ-treated HepG2 cell cultures visualized by optical microscopy 20X. (**B**) OLZ-treated cells showed a significant increase in total lipid content. (**C**) Neutral lipids quantification by Nile red and (**D**) polar lipids quantification by Nile Red * *p* < 0.05 vs. CTL (0 μg/mL). (**E**) The heatmap shows the normalized gene expression levels for 65 differentially expressed genes found when comparing OLZ and DG treatments. Expression for the two replicates of control (Control 1 and Control 2), OLZ-treated (OLZ1 and OLZ2), DG-treated (DG1 and DG2), and OLZ + DG-treated (OLZxDG1, OLZxDG2) cells are shown. Gene expression values were normalized by row using z-scores (**F**). The Volcano plot shows genes differentially regulated when comparing OLZ and DG treatments. Differentially expressed genes (FDR < 0.05) are shown as purple (higher expressed by DG) and yellow (higher expressed by OLZ) circles. Names are indicated for those genes showing at least a 1.5-fold expression difference between treatments. Circles in grey show genes with no differential expression.

One of the most important metabolic functions of the liver is maintaining glucose homeostasis through regulation of the lipid content in organisms. In this regard, an important marker of liver metabolic status is lipid accumulation. The effect of OLZ on HepG2 cells’ lipid content was studied using the dye Oil Red O (for total intracellular lipids, Figure 2A) and Nile Red, a fluorescent probe that allows the detection of polar and neutral intracellular lipids [26]. OLZ-treated HepG2 cells were stained and observed under an optical microscope, showing a significant increase in intracellular lipid droplets, compared to control counterparts (Figure 2A). These results demonstrate that OLZ induces a significant accumulation of intracellular lipids in liver cells in vitro (Figure 2B). In order to confirm these results obtained using Oil red O staining, we used Nile Red, a fluorescent probe previously validated as a marker for lipid accumulation in hepatocytes [27]. Our results show that a 50 μg/mL concentration of OLZ significantly increased intracellular neutral and polar lipids levels in HepG2 cells (Figure 2C,D). Recent studies have also reported an increase in oil red dye in hepatocytes of olanzapine-treated rats, together with an increase in triglycerides [28]. Differential gene expression was assessed in untreated cells and those treated with DG, OLA, or a combination of both. At a false discovery rate of 0.05, we found few genes were differentially regulated when comparing treatments to the control condition. Specifically, we found one gene downregulated by DG (CENP-E), two genes upregulated by OLA (GDF-15 and CAP20), and three genes upregulated by the OLA + DG combination (GDF-15, ACAS2, and CL6). This suggests a mild effect upon differential gene expression of every single treatment compared to the control condition. However, when performing a direct comparison between DG and OLA treatments, we found 65 differentially regulated genes (FDR < 0.05), 52 of which were highly expressed in OLA-treated cells, demonstrating a differential treatment response between drugs since a significant difference was observed in genes coding for enzymes that participate directly or indirectly in the synthesis of cholesterol and that are related to the signaling path of the SREBPs (Table 1 and Figure 2E,F).

### 2.3. Effect of DG and DS over Lipid Accumulation in HepG2

**Figure 3:**
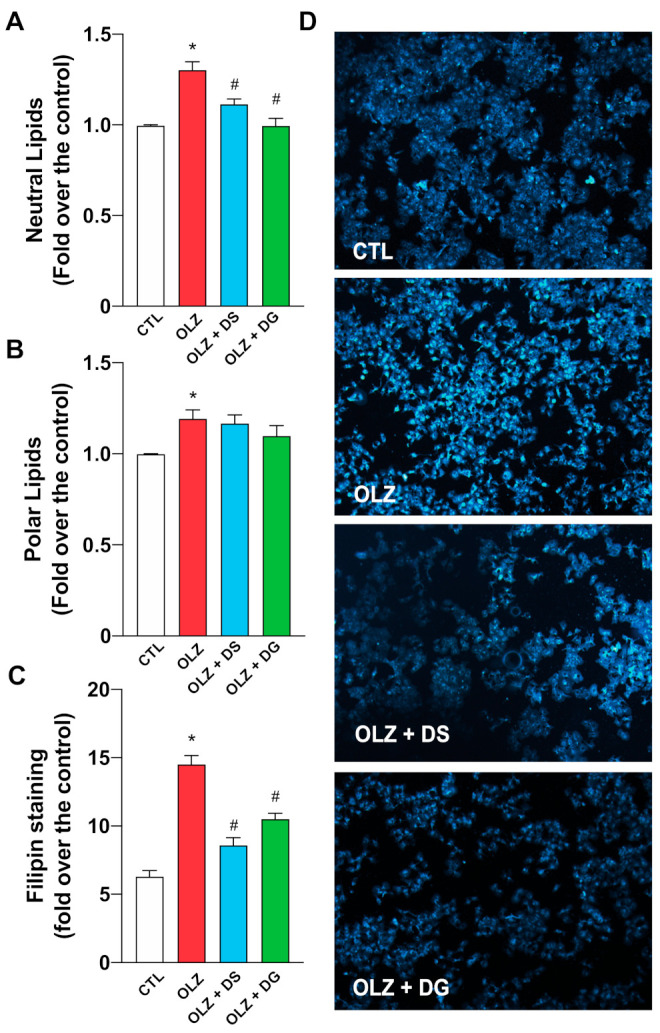
Response of DG and DS over lipid accumulation in HepG2. (**A**) Cells treated with OLZ plus DS or DG show a significant decrease in the accumulation of neutral lipids. (**B**) Cells treated with OLZ plus DS or DG did not show a protective effect against the accumulation of polar lipid induced by OLZ (**C**) DS, and DG prevented the accumulation of unesterified cholesterol induced by OLZ. * *p* < 0.05 vs. CTL. # *p* < 0.05 vs. OLZ (**D**) Representative images were visualized by fluorescence microscopy with a 10X objective. White bars represent control (CTL); red bars represent olanzapine (OLZ)-treated cells, blue bars represent the olanzapine and delphinidin-3-sambubioside-5-glucoside (DS) treatment, and green bars represent the olanzapine and delphinidin-3,5-diglucoside (DG) treatment.

Since Nile Red exhibits yellow fluorescence when it is dissolved in neutral lipids, and red fluorescence when dissolved in polar lipids, we observed alterations in lipid accumulation with differences in lipid polarity [26]. When HepG2 cells were co-incubated with OLZ 50 μg/mL for 24 h and DG or DS, Maqui anthocyanins produced a significant decrease in the accumulation of neutral lipids in HepG2 cells treated with OLZ (Figure 3A). In contrast, no protective effect by DG and DS against polar lipid accumulation was observed (Figure 3B), suggesting that the protective effect of DS and DG against OLZ-induced hepato-steatosis is based on the reduction of neutral lipids, such as acylglycerols, cholesterol, and cholesterol esters. In order to confirm the changes in polar lipids, we determined the impact of OLZ and anthocyanins on total and free cholesterol levels using Filipin, a fluorescent probe used for the detection of cholesterol. Our results show an increase in Filipin fluorescence in HepG2 cells treated with OLZ compared to the control group (Figure 3C,D), suggesting an OLZ-mediated increase of intracellular cholesterol in HepG2 cells. Interestingly DG and DS prevented the accumulation of unesterified cholesterol induced by OLZ, suggesting that cholesterol accumulation is key for the hepato-steatosis induced by OLZ and Maqui anthocyanins (DS and DG) could protect liver cells from this metabolic side effect.

### 2.4. Metabolic Effects of Olanzapine in Skeletal Muscle Cells

**Figure 4:**
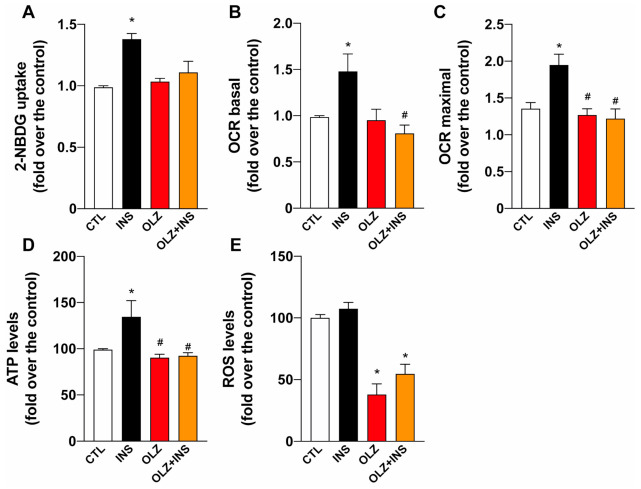
Metabolic effects of olanzapine treatment in skeletal muscle cells. (**A**) Glucose uptake determination by 2NBDG uptake. (**B**) The basal oxygen consumption rate (OCR) increased in L6 cells after insulin 100 nM stimuli. Incubation with OLZ decreased the insulin response. (**C**) Maximal OCR was determined after the addition of FCCP to uncouple oxidative phosphorylation. Insulin treatment resulted in an increase of maximal OCR, which was repressed in OLZ-incubated myoblasts. (**D**) ATP levels increased after insulin treatment, which was ameliorated by the incubation with OLZ. (**E**) ROS production was determined by DHR and was significantly decreased by OLZ. * *p* < 0.05 vs. CTL. # *p* < 0.05 vs. OLZ. White bars represent control (CTL) cells (no treatment), Black bars represent cells treated with insulin (INS), Red bars represent olanzapine-treated cells. Orange bars represent the OLZ + INS, which considered both treatments (olanzapine and insulin).

One of the most effective markers of metabolic syndrome in peripherical tissues is insulin resistance. We verified that insulin-induced glucose uptake is disrupted in L6 skeletal muscle cells by OLZ [36]. Our results show that after a 30-min INS stimulus, the uptake of the fluorescent glucose analog, 2NBDG, was significantly higher in control cells than in cells treated with OLZ. The incubation with 50 μg/mL -OLZ for 24 h impaired INS-induced glucose uptake without changing the basal glucose uptake (Figure 4A). Our results show that when cells were stimulated with 100 nM INS, the pre-incubation with OLZ impaired INS-induced mitochondrial boost by inhibiting oxygen consumption in basal and maximal conditions, causing impairment in the INS-induced mitochondrial response (Figure 4B,C). Moreover, insulin-induced ATP production is also impaired after incubation with OLZ in L6 skeletal muscle cells (Figure 4D). OLZ has been previously described as a potent ROS scavenger, and controversial effects on ROS production have been reported [37,38,39,40]. In this regard, we used the DHR fluorescence probe to determine ROS production after OLZ incubation and the possible effects on insulin-induced ROS signaling. Our results show that OLZ significantly reduced ROS production in L6 cells (Figure 4E).

### 2.5. Effects of Anthocyanins in Skeletal Muscle Cells

**Figure 5:**
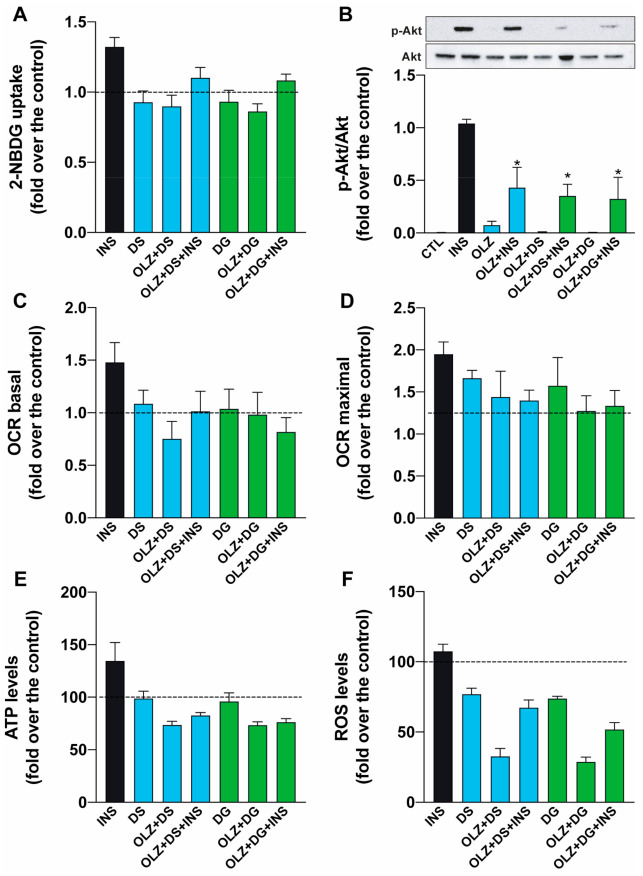
Effect of anthocyanins (DS and DG) co-incubated with OLZ after insulin response in L6 myoblasts. (**A**) Glucose uptake was measured after 24-h OLZ treatment in the presence (+) or absence (-) of DS or DG. Glucose uptake only increased in control cells (no treatment) after 30 min of a single 100 nM insulin stimulus (black bar). (**B**) Akt phosphorylation was determined by Western blot and normalized by total Akt to quantify differences among the conditions. OLZ treatment decreased p-Akt in response to insulin, and DS and DG co-incubation with OLZ did not restore phosphorylation levels. Mitochondrial metabolism: (**C**) Basal oxygen consumption rate (OCR) increased in L6 cells after insulin 100 nM stimuli (black bar), and incubation with anthocyanins (DS blue bars and DG green bars) decreased insulin response. (**D**) Maximal OCR was determined after the addition of FCCP to uncouple oxidative phosphorylation. (**E**) ATP levels were significantly decreased in the presence of OLZ and could not be rescued by DS or DG. (**F**) ROS production was significantly decreased after incubation with OLZ and was not restored after incubation with anthocyanins. * *p* < 0.05 vs. CTL. Dotted line represents the control condition with no stimuli (before insulin treatment).

In order to analyze the potential of DS and DG to protect the insulin response in muscle cells after OLZ incubation, we incubated L6 cells with or without DG or DS co-incubation and determined glucose uptake as an endpoint measurement after a 30-min 100 nM INS stimulus. Interestingly, neither co-incubation with DS nor with DG restored INS-induced glucose uptake when cells were incubated with OLZ (Figure 5A). Moreover, our results show that DG and DS cannot restore Akt phosphorylation after insulin stimulus in OLZ-treated cells (Figure 5B).

Another study showed that OLZ impairs the mitochondrial network [41]. Thus, we tested whether DS and DG could restore or protect mitochondrial function and morphology from the metabolic toxicity of OLZ. The incubation with DG did not increase oxygen consumption (Figure 5C,D). When cells were pre-incubated with DG and stimulated with INS, oxygen consumption was not significantly different from control cells. Moreover, when cells were co-incubated with OLZ plus DG for 24 h and INS-stimulated for 3 h, oxygen consumption did not increase (Figure 5C). Additionally, when the maximal oxygen consumption rate was measured, results were similar to basal consumption, and ATP production (Figure 5E) was also impaired after an INS stimulus, when L6 muscle cells were incubated with OLZ plus DG (Figure 5D), suggesting that the INS-promoted increase in mitochondrial metabolism is compromised and cannot be restored by DG. As the results above have described that the alterations in mitochondrial function and morphology in response to insulin continue to occur in the presence of olanzapine and anthocyanins (DS and DG), we tested a possible mechanism that could be involved in the prevailing effects of OLZ. Our results show that anthocyanins reduce ROS levels (Figure 5F). Interestingly, OLZ plus the anthocyanins showed a more significant decrease in ROS production than the anthocyanins (Figure 5F). Co-incubation with DS showed a similar response in mitochondrial function parameters and did not rescue the INS mitochondrial function response.

There is a close connection between mitochondrial function and mitochondrial morphology. In fact, L6 cells with forced mitochondrial fragmentation have shown altered patterns of mitochondrial function and INS signaling [41]. Moreover, OLZ has been shown to promote mitochondrial fragmentation, while insulin promotes mitochondrial fusion to improve mitochondrial metabolism [36]. In our model, OLZ induced mitochondrial fragmentation, which could still be observed when OLZ was co-incubated with DS or DG (Supplementary Materials Figure S1). This may be linked to the absence of response to DG and DS of L6 cells in mitochondrial function after OLZ incubation.

Our results confirm that OLZ impairs INS-induced glucose uptake in skeletal muscle and produces lipid accumulation in HepG2 cells, suggesting a metabolic disorder in L6 and HepG2 cell lines, which can be corrected only in liver cells but not in muscle cells.

## 3. Discussion

Metabolic syndrome is among the most problematic side effects of SGAs. Despite the efficacy of SGAs in the treatment of psychotic disorders, their metabolic toxicity decreases compliance with therapy [1]. Unfortunately, antidiabetic drugs in patients under SGAs treatment display limited efficacy in metabolic control [1]. Therefore, the study of potent antidiabetic polyphenols has emerged as a feasible path to developing a coadjutant strategy. More specifically, natural anthocyanins were previously reported as potent protective molecules against obesity and T2D [17,18,42]. Clinical data shows that dietary intake of anthocyanins and berry fruits has been consistently associated with a lower T2D risk [5,24,43]. A recent publication found that purified anthocyanins administered for a relatively short time (12-week) to pre-diabetic patients or early untreated diabetic patients improved glycemic control and the lipid profile [44]. Therefore, there is a solid rational framework to test whether natural anthocyanins could serve as lead compounds to develop coadjutant therapies against SGAs-induced T2D and insulin resistance. Since delphinidin-3-sambubioside-5-glucoside (DS), a natural anthocyanin particular to Maquiberry fruit (*Aristotelia chilensis*), showed a potency equivalent to metformin in decreasing glucose production in liver cells and displayed insulin-like effects in liver and muscle cells [18], DS is a plausible candidate against insulin resistance and metabolic toxicity induced by OLZ in skeletal muscle and liver. For this aim, we used a cellular model of skeletal muscle and hepatic cells to test whether the purified anthocyanins, DS and DG, inhibit insulin resistance and hepato-steatosis induced by OLZ. As has been previously reported, DG-enriched fractions of Maqui fruit and blueberries ameliorate insulin resistance in a high-fat diet-induced metabolic syndrome murine model [17,24] and DS (at a 100 μg/mL dose) increases insulin-mediated glucose uptake in cultured L6 muscle cells [18], thus unveiling a potential scenario for the use of anthocyanins in metabolic syndrome. Our results show differential effects in the hepatic and skeletal muscle models in response to anthocyanins. Specifically, neither DS nor DG increased the uptake of 2-NBDG in OLZ-treated skeletal muscle cells. Anthocyanins did not rescue Akt phosphorylation in skeletal muscle cells, which has been suggested by other studies to be one of the targets of olanzapine-induced insulin resistance [45]. However, DS and DG did decrease lipid accumulation in liver cells, which is also known to involve the Akt pathway [28]. We also observed that neither DS nor DG could protect or rescue skeletal muscle cells from OLZ-induced mitochondrial dysfunction. On the other hand, DS and DG rescued HepG2 cells from OLZ-induced lipid accumulation, suggesting differential effects of anthocyanins in each cell type. These observations led us to hypothesize that the accelerated T2D and INS resistance induced by SGAs would be mediated by a different set of mechanisms from those of diet-induced T2D. Perhaps SGAs-induced insulin resistance involves alterations in the pro-oxidants/antioxidants intracellular ratio, because olanzapine has been reported to have strong antioxidant properties and it induces mitochondrial fragmentation [36], which is linked with impaired mitochondrial dynamics and metabolic homeostasis.

Our hypothesis is confirmed by a recent study reporting that metformin fails to prevent OLZ-induced metabolic syndrome in rodents [46]. Remington et al. concluded that metformin attenuates hepatic insulin resistance observed with acute OLZ administration but fails to improve peripheral insulin resistance. Moreover, our results suggest that the potent antioxidant effects of OLZ could be responsible for the weak response of skeletal muscle cells to insulin. Based on the current literature and our results, we propose that the efficiency of GLUT-4 transporters’ translocation depends on the ROS production inside skeletal muscle cells, and antioxidants, like OLZ, may induce a decrease in GLUT-4 translocation [47]. It is feasible that the synergistic effect of OLZ and potent antioxidants (DG and DS) would contribute to the olanzapine-mediated impairment of GLUT-4-dependent glucose uptake in skeletal muscle cells. Meanwhile, DS and DG would display a beneficial effect in liver, through the decrease of OLZ-induced lipid accumulation. These differential effects in skeletal muscle and liver cells also suggest that there are direct effects of OLZ over each cell type that could be acting together with the inflammatory hypothesis [48,49].

## 4. Materials and Methods

### 4.1. Chemicals and Reagents

Culture medium Roswell Park Memorial Institute (RPMI) 1640, Dulbecco’s modified Eagle culture medium (DMEM), fetal bovine serum (FBS), antibiotic-antifungal, phosphate-buffered saline (PBS), trypsin, and ethylenediaminetetraacetic acid (EDTA) were purchased from Gibco™ (Waltham, MA, USA). Nile Red (C_20_H_18_N_2_O_2_), Oil Red O (C_26_H_24_N_4_O), dimethylsulfoxide (DMSO), Philippine, and paraformaldehyde were purchased from Sigma-Aldrich (St-Louis, MO, USA). Trizol reagent was obtained from Ambion^®^ (Waltham, MA, USA). Fluoromount-G was purchased from Southern Biotech (Birmingham, AL, USA & Canada), and Amplex Red Cholesterol Assay Kit was obtained from Invitrogen™ (Eugene, OR, USA). Delphinidin-3-sambubioside-5-glucoside (DS) and delphinidin-3,5-diglucoside (DG) were isolated from Maqui berries. Olanzapine Zyprexa^®^ injectable solution 10 mg/mL from Laboratorios Eli lilly was used.

### 4.2. Extraction, Isolation, and Characterization of Anthocyanins from A. chilensis

Maqui fruits (500 g) were extracted twice using 3 L of formic acid-ethanol solution (5:95, *v*/*v*) for 48 h under agitation. The crude extract was filtered under vacuum using a glass funnel filter with a sintered glass disc. Extracts were gathered and concentrated under reduced pressure and low temperature (<40 °C) and then freeze-dried (−55 °C for 36 h) to obtain 121 g of crude extract. Five grams of this extract were dissolved in 250 mL of acidified water (5% *v*/*v* formic acid) and loaded onto a glass column (40 mm i.d. × 300 mm) packed with Amberlite XAD-7 resin (Rohm and Haas, Chauny, France), previously conditioned with acidified water (5% *v*/*v* formic acid). The column was washed with 3 L of water at a flow rate of 10 mL min^−1^, and elution was performed with 1.5 L of formic acid-ethanol solution (5:95 *v*/*v*). This solution was concentrated under reduced pressure and then freeze-dried, giving a yield of 1.9 g. From this purified extract, DG and DS were isolated by centrifugal partition chromatography (CPC) employing an Armen Glider (Saint-Ave, France) centrifugal partition chromatograph Spot-CPC-250B Bio-extractor (SCPE) with a cell volume of 250 mL. SCPE was connected to an Armen SPOTPREP II system, equipped with an injection valve (10 mL loop), UV detector, and fraction collector. Separation was performed using a two-phase solvent system MTBE/n-BuOH/ACN/water (2:2:1:5 *v*/*v*/*v*/*v*) acidified with 0.1% TFA, as described elsewhere [50]. The CPC rotor was first filled with 1.5 column volumes using the upper phase at 30 mL min^−1^ and 500 rpm rotation. The lower phase was pumped into the system in descending mode at a flow rate of 12 mL min^−1^, increasing the rotation speed up to 2000 rpm. Purified extract (500 mg) was dissolved in 10 mL of 1:1 mixture of the upper and lower phase and loaded through a 10 mL sample loop. Fractions (25 mL, 27 tubes) were collected and monitored by scanning from 200 to 600 nm and fixed wavelengths of 280 and 520 nm. Extrusion was performed after 150 min with 100% stationary phase, increasing the flow rate at 30 mL min^−1^ for 10 min. The yields obtained were 58 mg of DG and 27 mg of DS with a purity of 97.6%, and 98.8%, respectively. Both compounds were analyzed by liquid chromatography tandem mass spectrometry (LC/MS/MS) using a Shimadzu UHPLC-DAD-ESI-MS/MS system composed of LC-30AD pump, DGU-20A5R degassing unit, SIL-30AC autosampler, CTO-20AC column oven, CBM-20A communication module, SPD-M20A DAD, and LCMS-8030 triple quadrupole (TQ) mass spectrometer. Chromatography was carried out on Phenomenex (Torrance, CA, USA) Kinetex XB core-shell C18 column (100 mm × 2.1 mm, S-1.7 µm) connected to a Kinetex guard column, both set at 35 °C, using a binary mobile phase composed of acidified ultrapure water (A, 5% *v*/*v* formic acid) and neat acetonitrile (B). The following gradient program was applied at a flow rate of 0.4 mL min^−1^: 0.1–3 min 2–15% B; 3–4 min 15–80% B; 4–5 min 80–80% B (isocratic step); 5–6 min 80–2% B and 6–10 min 2% B (column conditioning). MS analysis was carried out using the following parameters: ESI in positive mode, capillary voltage 3.0 kV, nebulizing gas (N_2_) 3 L min^−1^, drying gas (N_2_) 15 L min^−1^, desolvation line temperature 250 °C, and block temperature 400 °C. Mass spectra were acquired in full scan mode between *m*/*z* values of 50 and 1000. MS/MS analysis in product ion scan mode was performed using argon as the collision gas and voltage of −35 V. Data were acquired, recorded, and analyzed by means of Shimadzu LabSolution 5.8 software.

### 4.3. Cell Culture

The HEPG2 human hepatocarcinoma cell line (ATCC, (Manassas, VA, USA), HB-8065 TM) was cultured in Gibco^®^ RPMI 1640 culture medium supplemented with 10% FBS and 1% Gibco^®^ antibiotic-antifungal was used and maintained at 37 °C and CO_2_ 5% [50]. L6 (ATCC^®^ CRL-1458™) skeletal muscle cell lines were cultured in alpha minimal essential medium (α-MEM) (Gibco) supplemented with 10% fetal bovine serum (FBS), 1% non-essential amino acids, and 1% antibiotic-antimycotic mixture, in humidified air containing 5% CO_2_ at 37 °C. After two days, cells were cultured with α-MEM supplemented with 2% FBS [41].

### 4.4. Pharmacological Treatments

For experimentation, L6 cells were incubated with olanzapine 50 μg/mL (OLZ) for 24 h to simulate a chronic exposure in experimental conditions. For the insulin (INS) groups, the last 3 h were incubated with 100 nM INS [41]. Further, 50 μg/mL DG or DS were co-incubated with OLZ for 24 h when indicated or left untreated (control, CTL).

### 4.5. Total Lipid Staining with Oil Red O

After 24 h of treatment at 37 °C, cells were fixed with paraformaldehyde 4% for 15 min and marked with the Oil Red O dye as described previously [50]. Finally, cells were washed with 1X PBS, the remaining dye removed, and observed by optical microscopy with a 20X objective. Microphotographs were taken with the AmScope software (Irvine, CA, USA), and the colored area was determined with ImageJ software (Bethesda, MD, USA).

### 4.6. Nile Red Staining Determination by Flow Cytometry

Cells were released with Trypsin 1X and washed by centrifugation. Cells were incubated with Nile Red 0.25 μg/mL for 15 min, and fluorescence was measured with a BD Accuri C6 flow cytometer (BD, Oxford, UK). A 488 nm laser was used [27], and the living cell populations were selected from fluorescence emission measurement with the CFlow Plus program (Becton, Dickinson and Company, Franklin Lakes, NJ, USA). The use of Nile Red as an efficient marker for lipid accumulation cell culture and flow cytometry applications has been described elsewhere [27].

### 4.7. Glucose Uptake Determination by Flow Cytometry

Cells were washed 3 times with Krebs buffer without glucose and incubated with a fluorescent glucose analog, 2-deoxy-2-((7-nitro-2,1,3-benzoxadiazol-4-yl glucose) amino) (2NBDG), for 15 min. Then, cells were washed in Krebs Buffer with glucose and released with Trypsin 0.05% Gibco^®^ EDTA 1X, incubated for 3 to 5 min at 37 °C, 5% CO_2_ following the manufacturer’s instructions. Cells were centrifuged at 2000 rpm for 7 min. The supernatant was removed, and cells were resuspended in PBS 1X. Fluorescence was measured in a BD Accuri C6 flow cytometer. Laser 533/30 nm was used. This experimental analysis has been previously validated in [51] and also used in [52].

### 4.8. ROS Production Determination by Flow Cytometry

Cells were washed 3 times with Krebs buffer without glucose and incubated with the dihydrorhodamine 123 (DHR) probe. Then, cells were washed in Krebs Buffer with glucose and released with Trypsin 0.05% Gibco^®^ EDTA 1X, and incubated for 3 to 5 min at 37 °C, 5% CO_2_. Cells were centrifuged at 2000 rpm for 7 min. The supernatant was removed, and cells were resuspended in PBS 1X. Fluorescence was measured in a BD Accuri C6 flow cytometer. Laser 533/30 nm was used. This experimental analysis has been previously validated in [53].

### 4.9. Filipin Staining

After 24 h of treatment, cells were fixed with paraformaldehyde 1% for 20 min and dyed with Filipin for 30 min [54]. Finally, coverslips were adhered to the support with Fluoromount-G mounting medium for 1 h and observed with an Olympus bx50 fluorescence microscope (Tokyo, Japan). Cell images were collected with Q-Capture software and analyzed by ImageJ.

### 4.10. RNA-Seq and Statistical Analysis

Sequencing data was analyzed using the Galaxy platform [55] (http://www.usegalaxy.org, accessed on 7 September 2021, Bioproject Accession PRJNA762573.). First, high-quality sequencing reads were mapped to the human hg38 reference genome using the TopHat package (v 2.1.0, [56]) and genomic features from the human gencode annotation v27 [57]. Read counts per gene were calculated using the package featurecounts (v 1.4.6, [58]). Differential expressions between treatments were calculated using the DEseq2 package (v 1.14.1, [59]), for which Wald statistic *p*-values were adjusted with the Benjamini–Hochberg procedure to control for the false discovery rate (FDR). Genes with FDR < 0.05 were considered as differentially expressed. Data can be accessed in Bioproject Accession PRJNA762573.

### 4.11. ATP Measurements

Intracellular ATP content was determined using a Cell Titer-Glow Luminescent Cell Viability Assay (Promega) following the manufacturer’s instructions, as described elsewhere [60]. Signals were measured in a Tecan Infinite M200 Pro plate reader (Tecan, Switzerland).

### 4.12. Oxygen Consumption Rate Measurements

Oxygen consumption of L6 cells submitted to the different pharmacological treatments was determined at 30 °C in Clark’s electrode as extensively described by Kuznetzov et al. [61].

### 4.13. Western Blot Analysis

Cells were washed with cold PBS and lysed using NP40 (SIGMA-Aldrich, St. Louis, MO, USA). Lysates were centrifuged at 10,000 rpm for 10 min at 4 °C. Proteins were separated on SDS-PAGE polyacrylamide by molecular weight and transferred to PVDF membranes. Transferred membranes were blocked with 5% low-fat milk (Svelty, Nestlé) in TBS-T. Membranes were incubated overnight at 4 °C with primary antibodies p-Akt1/2 and total akt1/2 (Cell Signaling, Danvers, MA, USA) as described previously in [41].

### 4.14. Statistical Analysis

Data are presented as mean ± SEM of the indicated sample size (n). Multiple groups were analyzed using one-way ANOVA followed by a protected Tukey post-test. The GraphPad Prism 6 statistical program (GraphPad Software 2365 Northside Dr. Suite 560 San Diego, CA, USA) was used, and a *p*-value < 0.05 was considered statistically significant.

## 5. Conclusions

Our results suggest that anthocyanins modify lipid metabolism and rescue the OLZ-induced steatosis but do not influence insulin impairment induced by OLZ in skeletal muscle. Altogether, the current evidence suggests that Maqui anthocyanins have differential effects that could be dependent on the metabolic status of each tissue and on the primary source of energy of each cell type. It does not escape to our attention that our results might also be explained by changes in glucose homeostasis in the liver and skeletal muscle mediated by gluconeogenic signaling that could be altered with the use of SGAs. Moreover, it is suitable to propose that the strong antioxidant effect of anthocyanins, DS and DG, would synergistically contribute to the olanzapine-mediated impairment of GLUT-4-dependent glucose uptake in skeletal muscle cells. Therefore, the use of potent antioxidants in SGA users should be carefully evaluated.

## Figures and Tables

**Table 1:** Genes involved in the hepatic-steatosis induced by olanzapine and the protective effect of Maqui anthocyanins in HepG2 cells.

Gene	Function
ACSS2	Its expression is controlled by SREBPs and regulates the synthesis of Acetyl CoA from acetate (previous step from cholesterol synthesis) [29]
INSIG1	Insulin-regulated protein (INSIG) interacts with SCAP and HMG-CoA reductase and regulates SREBP activity [30]
MVD	Catalyze the conversion of mevalonate pyrophosphate into isopentenyl pyrophosphate [31]
LSS	Catalyze the conversion of (S) 2,3-oxidoesqualene to lanosterol [32]
TM7SF2	Participates in the conversion of lanosterol to cholesterol [33]
DHCR7	Catalyze the conversion of 7 dihydrocholesterol to cholesterol [34]
FASN	Regulates the synthesis of fatty acids [35]

## Data Availability

Not applicable.

## Supplementary Materials

The following are available online, Figure S1: Effects of Anthocyanins in OLZ induced mitochondrial fragmentation.

## Sample Availability

Samples of the compounds delphinidin-3,5-*O*-diglucoside (DG) and delphinidin-3-*O*-sambubioside-5-*O*-glucoside (DS) are available from the authors.
