# Supplementary material for: Anthocyanins from Aristotelia chilensis Prevent Olanzapine-Induced Hepatic-Lipid Accumulation but Not Insulin Resistance in Skeletal Muscle Cells
Source: Molecules. 2021 Oct 12;26(20):6149. doi: 10.3390/molecules26206149 (PMC8537850; doi:10.3390/molecules26206149)
Supplement: Supplementary file 1 [file molecules-26-06149-s001.zip › molecules-1391349-supplementary.pdf]

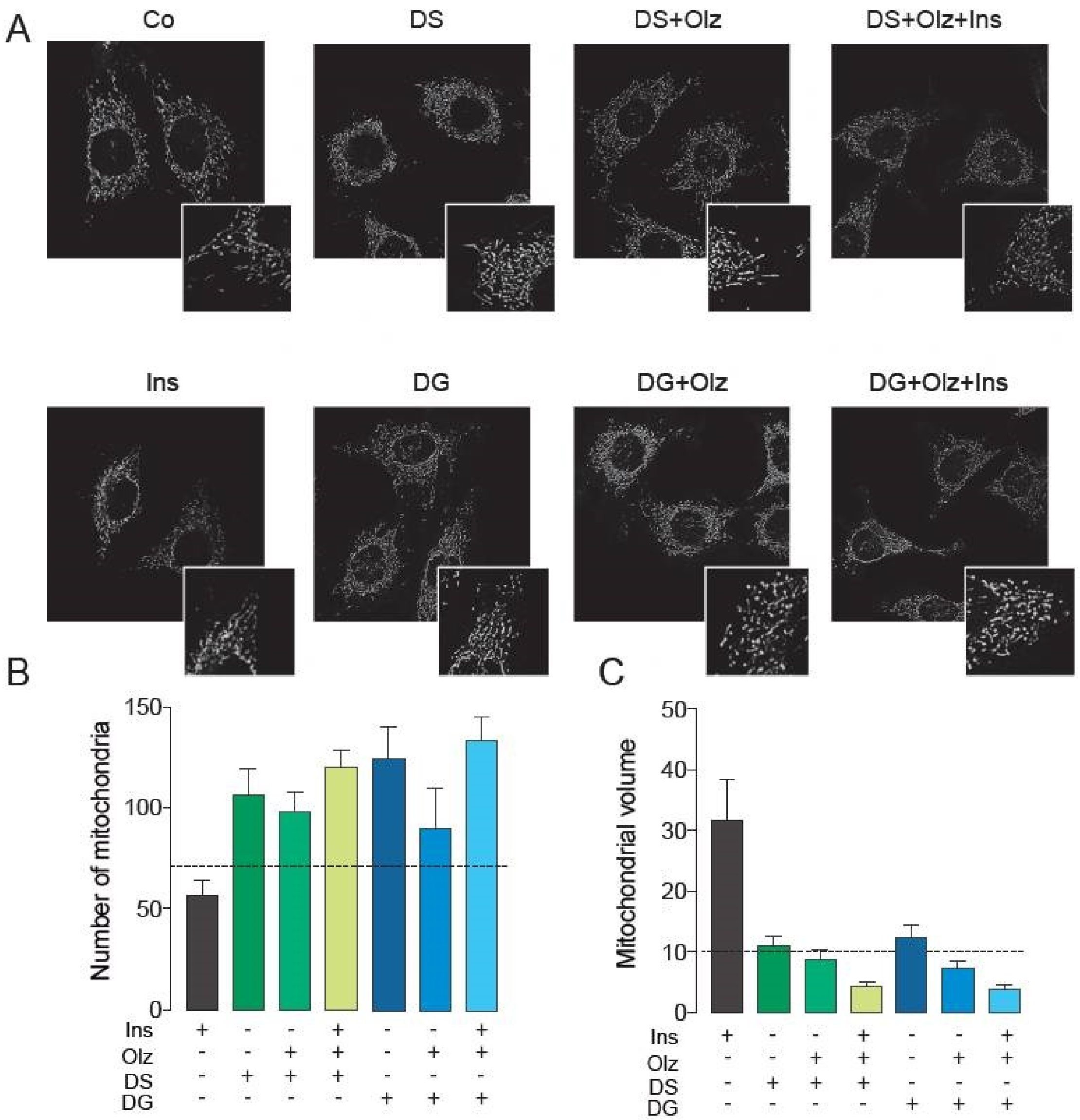

Supplementary Figure S1. Mitochondrial morphology analysis by confocal microscopy. (A) Representative images of L6 myoblasts incubated with Mitotracker green after the different treatments. Control. Insulin 100 nM. DG. DS. DG+ OLZ. DS+ OLZ. DG+OLZ+Ins. DS+OLZ+INS. (B) Quantification of the number of mitochondria per cell by Image J NIH software. OLZ treatment promoted an increase in the number of mitochondria that was also noted with the treatment of DG amd DS and in co-incubation. (C) Quantification of mean mitochondrial volume by Image J NIH software.
